# Deciphering the Microbiome: Integrating Theory, New Technologies, and Inclusive Science

**DOI:** 10.1128/msystems.00583-22

**Published:** 2022-09-08

**Authors:** Kathryn C. Milligan-McClellan, José Pablo Dundore-Arias, Jonathan L. Klassen, Ashley Shade, Linda L. Kinkel, Benjamin E. Wolfe

**Affiliations:** a Department of Molecular and Cell Biology, University of Connecticut, Storrs, Connecticut, USA; b Department of Biology and Chemistry, California State University Monterey Bay, Seaside, California, USA; c Institute for Systems Genomics, University of Connecticut, Storrs, Connecticut, USA; d Department of Microbiology and Molecular Genetics and Department of Plant, Soil and Microbial Sciences, Michigan State University, East Lansing, Michigan, USA; e Department of Plant Pathology, University of Minnesota, St. Paul, Minnesota, USA; f Department of Biology, Tufts University, Medford, Massachusetts, USA

**Keywords:** collaboration, ecology, evolution, integration, microbiome, technology, theory

## Abstract

The diversity and functional significance of microbiomes have become increasingly clear through the extensive sampling of Earth's many habitats and the rapid adoption of new sequencing technologies. However, much remains unknown about what makes a “healthy” microbiome, how to restore a disrupted microbiome, and how microbiomes assemble. In December 2019, we convened a workshop that focused on how to identify potential “rules of life” that govern microbiome structure and function. This collection of mSystems Perspective pieces reflects many of the main challenges and opportunities in the field identified by both in-person and virtual workshop participants. By borrowing conceptual and theoretical approaches from other fields, including economics and philosophy, these pieces suggest new ways to dissect microbiome patterns and processes. The application of conceptual advances, including trait-based theory and community coalescence, is providing new insights on how to predict and manage microbiome diversity and function. Technological and analytical advances, including deep transfer learning, metabolic models, and advances in analytical chemistry, are helping us sift through complex systems to pinpoint mechanisms of microbiome assembly and dynamics. Integration of all of these advancements (theory, concepts, technology) across biological and spatial scales is providing dramatically improved temporal and spatial resolution of microbiome dynamics. This integrative microbiome research is happening in a new moment in science where academic institutions, scientific societies, and funding agencies must act collaboratively to support and train a diverse and inclusive community of microbiome scientists.

## EDITORIAL

Microbiomes are communities of microbes, including bacteria, viruses, fungi, and microeukaryotes, that live together in a host or in an environment. Thousands of studies have documented the diversity of microbiomes across the globe and how they can control the biology of hosts and environments. Despite incredible progress in microbiome research over the past 2 decades, many questions remain about the structure and function of microbial communities.

In December 2019, we asked hundreds of researchers from across the US and other countries to convene for a 3-day, in-depth discussion about open questions in microbiome science and how to accelerate understanding in these areas. Participants spanned a wide variety of disciplines and career stages and met in a hybrid format, with both in person and online attendees (as described in [1]). We identified research priorities and open questions, speculated on technologies that are needed to advance these studies, and discussed whether general “rules of life” might be identified for microbiomes. Many of the themes from our discussions, major concerns that were raised, and potential future directions for microbiome research are included in this *mSystems* special collection “Deciphering the Microbiome” (https://journals.asm.org/topic/sss-taxonomy/special-series-msys-decmicro). Below we provide an overview of these pieces and other highlights from the meeting.

## TECHNOLOGIES AND TOOLS NEEDED

Major technological developments have led to dramatic advances in microbiome studies over the last few decades. In addition to the well-discussed and rapidly evolving sequencing platforms, microscopes have become more powerful, artificial environments have been created to conduct studies in highly controlled laboratory settings, and single-cell transcriptomics and microfluidic devices all allow for both large- and small-scale resolution of diverse interactions (2–4). However, in discussions at our workshop, several technologies were noted to be lacking, underdeveloped, or underutilized.

Microbiomes are complex communities, often numbering in hundreds of species. Many of these microbes have yet to be isolated or grown in laboratory studies, restricting the types of studies that can be performed to determine their roles in microbiomes or maintaining the health of the host or environment. Tools and reagents are needed to isolate more microbes to complete microbe collections. In addition, standardized methods and protocols to efficiently and reproducibly create complex communities and experimental conditions are generally lacking. This hinders the ability to perform, interpret, and compare experiments and to identify interactions among microbial members of the microbiome and between the microbiome and their hosts/environment.

The complexity of studying the interactions between microbiomes and their hosts and environments also requires a complex bioinformatic toolkit and an increased diversity of the people studying microbiomes. For example, although extensive tools are available for studying bacterial genomes, relatively fewer tools are available for fungi, protists, or viruses, resulting in a weaker understanding of the roles of those microbes in microbiomes (5, 6). Similarly, although toolkits have expanded for studying metabolites and 16S rRNA gene diversity within a microbiome system, few tools are available to study how these interact within the system, longitudinally over the lifetime of the host, or in conjunction with changes in the environment. Overcoming these challenges will require statisticians, bioinformaticians, computer scientists, and mathematicians to work collaboratively with microbiologists and microbiome scientists.

Several Perspective pieces in this series present new approaches for making sense of microbiome sequence data or new tools to fill major gaps in our knowledge of the diversity and functions of microbiomes. Tierney et al. rethink how we should analyze and categorize the deluge of microbiome sequence data (7). Much of our historical understanding of microbiology is based on microbes that can be cultured. However, high-throughput sequencing is rapidly expanding our understanding of the vast diversity of microbes. This piece argues that we cannot let databases and taxonomic frameworks based largely on culture-based microbiology constrain our computational approaches for making sense of microbiome sequence data.

New analytical approaches may also help bridge microbiome-affiliated disciplines and fill critical gaps in microbiome science. Microbial metabolites are key mediators of microbiome assembly and function, but accurate identification of all metabolites in microbiomes remain a challenge. Quinn et al. argue that one solution to this problem is better communication and collaboration between analytical chemists and microbiologists (8). Part of their proposed solution is simply having chemists and microbiologists spend more time working and learning together to understand the languages and methodologies of each of their distinct fields. Ongoing funding opportunities from the NSF and other federal agencies seek to address this problem by supporting cross-disciplinary workshops like ours and collaborative research grants. These authors also argue that new training paradigms are needed in academia to develop “chemoinformaticians” who can comfortably span the divide that often exists between chemistry and microbiology.

Once we have detailed data sets of the metabolites produced within microbiomes, modeling approaches can provide useful predictive frameworks to describe microbial interactions and microbiome functions. For example, in a Perspective piece, Ankrah et al. provide an overview of opportunities and challenges for the application of Genome-Scale Metabolic Models (GEMs) to microbiome research (9). Their piece highlights the potential of GEMs to generate testable hypotheses about the metabolic functioning of organisms growing in diverse environmental contexts, and subsequently, predict potential environmental changes driven by the metabolic activities within the community. The authors also outline community-identified barriers and resource gaps that must be filled to maximize the accessibility of modeling techniques and more transparent communication and interpretation of simulation results in microbiome research.

## EMERGING CONCEPTS AND THEORY

Although many microbiome studies have carefully characterized the diversity of microbial species within microbiomes, the processes that generate microbiome diversity are still being elucidated (10–13). Many conversations at our workshop and several pieces in this issue focused on identifying ecological and evolutionary concepts or theory that can help microbiome science better define how microbial communities assemble.

In this issue, Bittleson et al. explore questions of trait-based frameworks to inform microbiome assembly over space and time (14). In their piece, they propose expanding the yield-acquisition-stress (YAS) trait framework to both succession over time and biogeographic gradients to inform patterns of diversity. They use the simple pitcher plant system as a case study of how the YAS framework can be applied.

Also in this issue, the hot topic of community coalescence is discussed by Rocca et al. (15) Coalescence is the merging of microbiome members from different originating sources, as may happen when soil communities merge with lake communities after a large storm that generates runoff, or when wastewater effluent is merged into the groundwater or a stream. Coalescence can provoke new member interactions and a reorganization of general biotic context within a microbiome. Rocca et al. extend the coalescence framework to the goals of microbiome engineering and ask how predicting the outcomes of coalescing microbiomes may support applied goals in managed systems.

## INTEGRATION ACROSS SCALES

Another important theme that emerged at the meeting is the importance of scale in microbiome research. At what scales should we quantify microbiomes, hosts, and environments, and changes within and between those systems? What scales have been overlooked in previous microbiome studies? What types of data and analytical frameworks are needed to span spatial and temporal scales?

Beatty et al. provide one perspective on how to bridge multiple scales of microbiome complexity using remote sensing technologies (16). By integrating on-the-ground microbiome sequence studies with landscape and global-scale observations of microbiome processes from satellites and drones, scientists can now connect global-scale microbial phenomena with cellular-scale mechanisms. This piece argues that the fields of machine learning and spatial statistics are key to this data integration. Integration of these approaches to microbiome research is critical for effectively addressing global-scale microbial-mediated challenges, including climate change, pathogen outbreaks, and biodiversity loss.

The scale(s) at which we sample (e.g., large pieces of material that are homogenized) is often not the scale on which microbes interact. In another Perspective piece, Kashtan et al. argue that we can borrow concepts from economics to resolve this mismatch of scales (17). They point out that much of microbiome science is currently conducted in the same way as macroeconomics studies economies: we often consider properties of the whole system and rarely examine within-system heterogeneity. They argue that just as microeconomics considers how individual behaviors and other variations within systems impact economies, so too should microbiome scientists consider how within-system heterogeneity can explain whole-microbiome phenomena. By examining how macroeconomics and microeconomics work together to span economic scales, microbiome scientists can gain insights into how to bridge the various scales of microbial communities.

When trying to understand general principles that can translate across many different microbiome systems and scales, system-specific features and the myriad of microbiome methodologies can represent barriers. David et al. argue that deep transfer learning approaches can discover microbe-microbe and microbe-environment interactions that are generalizable across systems (18). Using approaches developed for natural language processing, these authors argue that deep transfer learning approaches can help discover a generalized lexicon of microbiomes that could be used across vastly divergent microbiome types and different scales of resolution. For those of us who have been overwhelmed when trying to understand what machine learning or neural networks can do for microbiomes, this Perspective is a great starting point.

## BUILDING AN INCLUSIVE COMMUNITY OF MICROBIOME SCIENTISTS

One of the most important themes to emerge from these conversations is how to build a diverse and inclusive community of microbiome scientists. What training opportunities are needed to prepare future microbiome scientists to tackle all the grand challenges outlined above? How do we get academic institutions, government agencies, and other organizations to work across disciplines to conduct truly integrative microbiome research? Who historically and currently lacks access to microbiome research and training opportunities? How have inequities, biases, and structural barriers shaped how microbiome science has been conducted and limited who has access to microbiome research?

Foxx et al. propose several ideas for how to improve access to microbiome science for previously excluded groups and how to create training opportunities for underrepresented groups. They also highlight how language and communication barriers have prevented access to microbiome conferences and other training opportunities. The Editorial team is especially grateful for the highly collaborative and integrative nature of this piece. In the spirit of building bridges across the many fields of microbiome science, we asked three separate teams representing diverse career stages and perspectives to work together on one synthetic piece. Their piece highlights how much work needs to be done to address the many challenges and inequities in microbiome science but also highlights many solutions that we can use when we step out of our disciplinary lanes and join together.

DeWolfe et al. argue that the human microbiome studies that use race as a primary variable to explain variations in microbiomes and their contribution to human health obfuscates the contributions of systemic inequities and other factors that may drive differences between populations (19). This is especially salient given that the variable “race” has no biological significance in human studies. They describe the history of race in microbiome studies, ghost variables that are the true variables behind the variable “race,” and how the use of race as a variable contributes to racism. This transdisciplinary team of a microbiologist, geographer, anthropologist, and evolutionary biologist also provides a roadmap for antiracist microbiome science.

## CONCLUSION

The 2019 “Deciphering the Microbiome” workshop and these associated Perspective pieces were instigated by the National Science Foundation’s “understanding the rules of life” initiative (20). This initiative proposes that there are sets of rules that predict a biological system's observable characteristics. NSF also emphasizes that these rules should span different scales of biology (space, time, levels of biological organization, etc.) and could be generalizable “beyond the system under investigation, so that a rule can be formulated”.

As we step back and view these Perspective pieces as a synthetic collection and reflect on discussion themes at the 2019 workshop, we can consider how rules of life might emerge in microbiome science by asking a few questions. How have other areas of microbiology or fields of science identified rules that provide generalizable and foundational knowledge for their disciplines? What concepts, tools, or analytical frameworks are necessary to identify these rules? What are current barriers for developing rules of life in microbiome science? What past advances in microbiome science helped provide general principles and how could future discoveries build on this foundational work?

As some of the Perspective pieces in our collection point out, reproducibility and standardization are essential for ease of comparing data sets to search for common patterns and processes. With a remarkable number of nucleic acid extraction techniques, sequencing approaches, and analytical platforms, the lack of standard microbiome methods has hampered our ability to build and share data sets where generalizable patterns can rise above methodological noise. Many recent calls for improving standardization of experimental systems, methods, and analyses are beginning to address this challenge (4, 21–24). For example, the National Microbiome Data Collaborative (NMDC) (25) seeks to facilitate transdisciplinary microbiome studies and collaborations by harmonizing and democratizing national resources for microbiome data exploration and sharing.

Microbiome scientists may not need to invent new rules to explain patterns and processes of microbiome diversity. As is highlighted by several papers in this collection, microbial ecologists have been successfully borrowing rules and theory from plant and animal ecologists that can often be modified to the unique biology of microbial systems (26–29). We could also borrow from outside biology, including math, chemistry, and other natural sciences. For example, thermodynamic and biophysical principles may be useful for understanding traits of soil bacteria (30).

For microbiome principles to be generalizable, they need to be applicable to diverse microbiome systems. One very strong message that emerged after our 2019 workshop was that many microbiome scientists tend to specialize in one or two systems and rarely interact with other scientists that study different microbiomes. For example, even though common ecological and evolutionary mechanisms may shape the structures of human and plant microbiomes, these research communities do not often go to the same scientific meetings or collaborate on research projects. There may be exciting commonalities across microbiomes that we could learn together if we had more interdisciplinary conversations and if research projects spanned many different systems. Fortunately, these divisions across microbiome science may be fading. Journals like *mSystems* and *Microbiome* encourage authors working on disparate microbiome systems to publish articles in collections with cross-cutting themes (31, 32). Funding agencies are increasingly encouraging researchers to ask questions that span multiple microbiome systems (33). Scientific meetings are also working to advance interdisciplinarity by organizing conference sessions that focus on common themes across very different microbiome systems (34). Similarly, integrated network efforts such as US Microbiome Centers Consortium (35) and the EU MicrobiomeSupport Consortium (36) have emerged as alternatives to facilitate communication and the establishment of synergistic collaborations and cross-pollination of ideas among microbiome scientists across multiple disciplines and institutions.

Rules can only be developed for microbiome systems where most of the key biological “parts” have been characterized. That is what much of the last 20 years of microbiome science has focused on, with many thousands of high-throughput sequence surveys of Earth’s microbial habitats. Efforts like the Human Microbiome Project and the Earth Microbiome Project helped collect data in a standardized way that can be used by many researchers to seek general patterns of microbial diversity (37–39). The baseline knowledge and standardized frameworks from these projects have facilitated even larger-scale and more in-depth standardized surveys of microbiomes (40–42). But there are still critical gaps in our understanding of the microbial “parts” in most microbiome systems. Many microbiome studies still focus only on a subset of the microbes present in a system (often just prokaryotes), either due to the use of primers that only target one microbial group or because databases to analyze metagenomic data are often biased toward prokaryotes. Viruses are also increasingly recognized as diverse and functionally significant players in most microbiome systems but are rarely characterized in the same studies as prokaryotic and eukaryotic microbes (43). New methods and approaches that capture a larger extent of complete microbiome diversity and functions are helping to address this grand challenge (44–46). How we train future microbiome scientists may also play a role in removing taxonomic blinders in microbiome science. If our trainees gain skills that can be universally applied to characterize the structures and functions of all microbial entities, they will be able to fully decipher the microbiomes of our planet.

There are many important themes from our workshop that we could not address in this small series of articles. Although this brief overview cannot fully capture the richness and diversity of the meeting discussions and Perspective pieces, we are excited to offer this collection of articles as a source of novel foundational ideas and insights that can further advance integrative microbiome research.
